# Life history and biocontrol potential of the first female-producing parthenogenetic species of *Diglyphus* (Hymenoptera: Eulophidae) against agromyzid leafminers

**DOI:** 10.1038/s41598-018-20972-3

**Published:** 2018-02-19

**Authors:** Fu-Yu Ye, Chao-Dong Zhu, Zoya Yefremova, Wan-Xue Liu, Jian-Yang Guo, Fang-Hao Wan

**Affiliations:** 1 State Key Laboratory for Biology of Plant Diseases and Insect Pests, Institute of Plant Protection, Chinese Academy of Agricultural Sciences, Beijing, 100193 China; 2 State Key Laboratory of Zoological Systematics and Evolution, Institute of Zoology, Chinese Academy of Sciences, Beijing, 100101 China; 3 Steinhardt Museum of Natural History, Department of Zoology, Tel Aviv University, Ramat Aviv, 69978 Israel

## Abstract

*Diglyphus* species are dominant biocontrol agents for suppressing outbreaks of agromyzid leafminers in fields. In July 2015, we collected a new thelytokous species of *Diglyphus* in Qinghai, China. The wasp is here named as *D*. *wani* Liu, Zhu & Yefremova sp. nov., based on morphological and molecular analyses. The life history and biocontrol potential of the wasp were studied in the laboratory and by providing *Liriomyza sativae* larvae. The intrinsic rate of increase, finite rate of increase, and mean generation time were 0.2373 d^−1^, 1.2678 d^−1^, and 15.9 d, respectively. The wasps showed three types of host-killing behaviour, namely parasitism, host feeding and host stinging, resulting in 47.6, 94.4 and 3.4 host larvae killed per wasp over a lifetime, respectively. The finite total host-killing rate was 1.0071 and the wasp showed strong synovigeny. The wasp may be a crucial biocontrol agent against agromyzid leafminers.

## Introduction

Agromyzidae is a dipteran family consisting of approximately 2,750 species^1^. Of these, ~110 species are known to be major pests of cultivated crops worldwide^2^. Leaf mining is generally the most widespread feeding behaviour, shared by more than 75% of the species^3^. The genus *Liriomyza*, which currently contain more than 330 species, is the most important and most extensively studied genus of Agromyzidae^3,4^. Among the *Liriomyza*, ~24 species are major economically important pests of agricultural and ornamental plants, and at least six species, *L*. *trifolii*, *L*. *sativae*, *L*. *huidobrensis*, *L*. *bryoniae*, *L*. *strigata* and *L*. *longei*, are polyphagous^4–6^. In China, at least 130 species of Agromyzidae have been reported, and six species, including the invasive species *L*. *trifolii*, *L*. *sativae*, *L*. *bryoniae* and *L*. *huidobrensis*, and the native species *Chromatomyia horticola* and *L*. *chinensis*, have caused serious damage to economically important crops, especially vegetables^5,7^.

Most economically important agromyzid leafminers are ecologically adaptable, exhibit strong polyphagy, leaf-mining activity, and reproductive capability, and are commonly resistant to insecticides^4,5^. Owing to these characteristics, they can easily establish new populations and are considered promising targets for biological control by hymenopteran parasitoids, which offer an alternative mechanism for limiting leafminers outbreaks^5,6^.

Prior to the 1950s, agromyzid leafminers were considered small-sized pests causing no significant economic losses in their areas of origin, and they got little attention because of the natural control by parasitic wasps^8^. With the frequent use and abuse of pesticides, the natural enemies of agromyzid leafminers were suppressed, and with increased commercial trade and anthropogenic activities, some agromyzid leafminers, especially some invasive *Liriomyza* species, gradually invaded and spread worldwide, resulting in serious crop damage in the invaded areas^5,6^. Thus, the management of agromyzid leafminers continues to be a topic of extensive research.

Agromyzid leafminers are subject to parasitoids, particularly in their areas of origin, which may regulate leafminers populations in pesticide-free areas^6,9–11^. Noyes^10^ listed more than 300 species of agromyzid parasitoids and over 80 species that attack *Liriomyza* species. Liu *et al*.^6^ reported that more than 140 species of parasitoids are known based on extensive and worldwide investigations of natural enemies of *Liriomyza* species. Musundire *et al*.^10^ calculated that 90 parasitoids, with species belonging to 10 families and 28 genera, were associated with only 20 agromyzid species belonging to 10 genera in the Afrotropical region. The comparatively low parasitoid diversity in this region may be reflected limited sampling effort and a lack of taxonomic expertise for parasitoid species^10^. Thus, the discovery and accurate identification of agromyzid parasitoids is important for their implementation biological control of agromyzid leafminer. Although chemical control is still used extensively, biological control by the rational release of parasitoids is a primary strategy for effective suppression of leafminers on field- or greenhouse-grown crops^6,7,12,13^. However, many parasitoid species that are dominant in fields still need to be extensively studied and, in particular, undergo biocontrol potential assessments.

*Diglyphus* (Eulophidae) is an economically important genus of solitary ectoparasitoids against agromyzid leafminers^7–16^, with a wide distribution wordwide^15^ (39 species). There are 15 and 16 species of *Diglyphus* distributing in Europe and China, respectively^7,14,17^. Occasionally, some *Diglyphus* species (e.g. *D*. *begini*, *D*. *chabrias*, *D*. *isaea* and *D*. *minoeus*), have also been reported on other hosts, such as Lepidoptera, Gracillariidae, Lyonetiidae, and Nepticulidae^17^. However, the ecological adaptability, biocontrol potential and application practices of *Diglyphus* species have mainly been studied in *D*. *isaea*, *D*. *begini*, *D*. *intermedius*, and mostly in *D*. *isaea*^7,18^. There has been limited biology and bio-control research on other species of the genus, even in those that are dominant in the field.

The female adult wasps of *Diglyphus* species show three types of host-killing behaviour, reproductive parasitisation (parasitism), non-reproductive host killing by feeding (host feeding), and host stinging without oviposition or feeding (host stinging), with the non-reproductive host-killing behaviour (host feeding and host stinging) to significantly increase the biocontrol potential^7,13,18–21^. For these species that express different types of host killing, we need to evaluate all of them separately and cumulatively to acquire information on their precise biocontrol potential^13,22–24^.

In July 2015, we first discovered an unidentified dominant species of *Diglyphus* attacking *C*. *horticola* that infests *Pisum sativum* in a field in Xining, Qinghai, China. This wasp only produces females without mating, and no males have been found in the field or subsequent laboratory colonies, which indicate that the species reproduces by thelytokous parthenogenesis (or female-producing parthenogenesis). No thelytokous species have been reported in the genus *Diglyphus* to date.

Hymenopteran parasitoids generally show haplo-diploid sex determination. Males are produced from unfertilised eggs and are haploid, whereas females develop from fertilised eggs and are diploid. In the Hymenoptera sexual reproduction is defined as arrhenotoky and asexual reproduction as thelytoky^25^. Female-producing parthenogenesis or ‘thelytoky’ refers to the production of female progeny from unfertilised eggs without mating^26^. Unisexual reproduction is not rare among parasitoids. Currently, according to our statistics, at least 210 species of thelytokous hymenopteran parasitoids are known. Some thelytokous wasps have been considered to be better biocontrol agents than arrhenotokous wasps because thelytokous reproduction can potentially lower costs in mass rearing facilities and increase parasitoid efficacy in the field^27,28^. Thus, the objectives of the present study were: (i) to identify the newly found thelytokous parasitoid species of *Diglyphus* using a combination of morphological features and molecular tools, because it is difficult to distinguish species from morphology alone owing to homoplasty among eulophids; and (ii) to evaluate the biocontrol potential of this thelytokous species on the basis of life history and measurements of parameter under laboratory conditions. The results will improve our understanding of the biological control efficacies of thelytokous parasitoids in the field.

## Results

### Species identification

#### Morphological diagnosis

**Figure 1:**
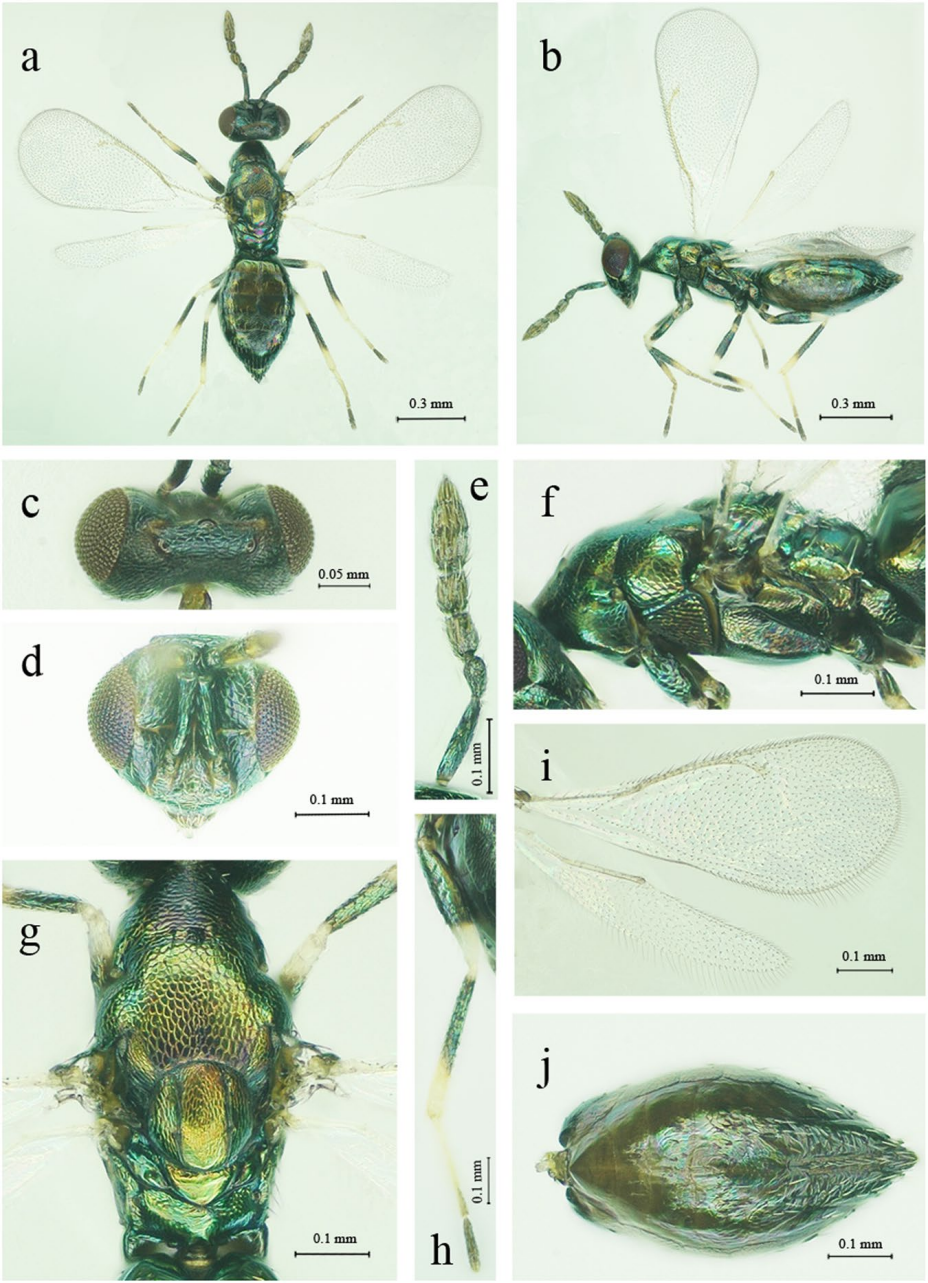
*Diglphus wani* sp. nov., female: (**a**) Body, dorsal view. (**b**) Body, lateral view. (**c**) Head, dorsal view. (**d**) Head, front view. (**e**) Left antenna, lateral view. (**f**) Mesosoma, lateral view. (**g**) Mesosoma, dorsal view. (**h**) Hind leg. (**i**) Right fore and hind wings. (**j**) Metasoma, ventral view.

**Figure 2:**
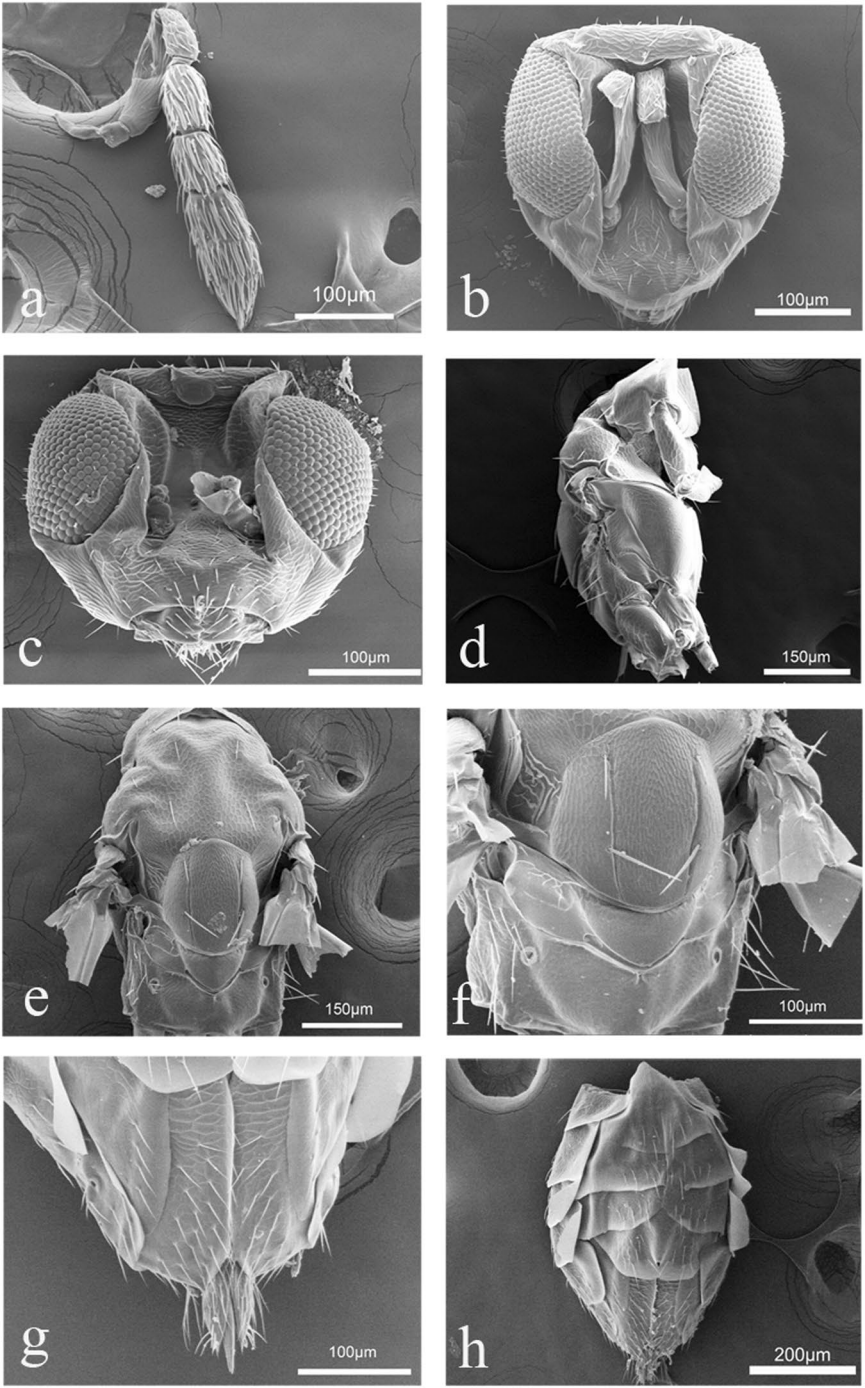
*Diglphus wani* sp. nov., female: (**a**) Antenna. (**b**) Head, frontal view. (**c**) Head, frontal view. (**d**) Mesosoma, lateral view. (**e**) Mesosoma, dorsal view. (**f**) Scutellum and propodeum, dorsal view. (**g**) Last sternite of gaster with sheaths of ovipositor, ventral view. (**h**) Metasoma, ventral view.

***D. wani*** **Liu, Zhu & Yefremova sp. nov**. (Figs 1 and 2).

**Holotype**: ♀ (IZAS, Institute of Zoology of Academy of Sciences, Beijing, China), China, Qinghai, (36°43′23.52″N, 101°45′1.51″E) collected from *Chromatomyia horticola*. Date, July 2015, Fu-Yu Ye and Shu-Long Lu.

**Paratypes:** 10 ♀ with same labelling date as the holotype (Institute of Plant Protection, Chinese Academy of Agricultural Sciences, Beijing, China). Additional paratypes (10 ♀) were deposited in the Institute of Zoology, Chinese Academy of Sciences, Beijing, China. 10 ♀ (IZAS, Institute of Zoology of Academy of Sciences, Beijing, China).

**Material examined**. 200 ♀ with same labelling date as the holotype and paratypes. 828 ♀ (reared from larvae of *L*. *sativae* in laboratory).

**Description**. Female. (Fig. 1a–h). Body length 1.00–1.85 mm, forewing length 0.92–1.20 mm. Colour: Body dark green with metallic tint; tegulae dark green, antenna brownish; mandibles yellow, labial and maxillar palpae pale yellow, compound eyes dark red. Legs with dark green coxae, brown trochanters, anterior 0.75 to middle of all femora dark brown, posterior pale yellow, all tibiae dark brown with metallic shine except base and apical 0.2–0.4 part white or pale yellow, black and white-yellow border on hind tibiae is gradient, tarsi yellow, except last 4th tarsomere (dark brown) and 3rd tarsomere (brownish), wings hyaline.

**Antenna** (Figs 1c and 2a). Brownish, 7 segments. Antenna with scape 3.3× as long as broad, pedicel 1.8 × as long as broad, 2 anellii, F1 1.5× as long as broad, F2 1.3× as long as broad, clava 3-segmented 2.3× as long as broad. F1 1.1× as long as F2, clava 1.2× as long as scape and 2.2× as long as F2. Scape cylindrical, metallic dark, with short setae and reticulation. All funicle concolorous. Clava with C3 with apical terminal spine. Flagellum with trichoid and capitate peg sensillae.

**Head** (Figs 1c,d and 2b,c). Head wider than height. Orthognathous. POL 2.7× as long as OOL. Eyes black-red with short setae. Frontal groove present, straight, running from 1/3 anterior margin of eyes. Vertex and frons with finely-meshed reticulation. Toruli inserted level with the lower margin of eyes. Malar sulcus present, straight, mouth 1.6× of malar space. Mandibles with 4 teeth. Maxillary palpus with 2 segments, labial palpus with 1 segment.

**Thorax** (Figs 1f,g and 2d,e,f). Pronotum shorter than mesoscutum, reticulate. Pronotum without transverse carina. Pronotum, mesontum and scutellum metallic green. Mesoscutum with notauli, with strong reticulation and areolae larger than that on scutellum, with 2 pairs of long setae on mid lobe. Mesoscutum 1.1× as long as scutellum. Scutellum as long as broad. Scutellum finely reticulate, with two straight sublateral grooves, with two anterior short setae and two posterior long setae. Dorsellum smooth. Propodeum 3.7× as broad as long, smooth, without median carina. Spiracle round, small with paraspiracular carina. Callus with 2 setae in first row and 2 setae in the second row.

**Wing** (Fig. 1i). Forewing hyaline, length 0.92–1.20 mm, broad 0.33–0.51 mm. Speculum present very small with sparse setations. Forewings 2.4× as long as broad. SMV tapering to apex, with 6 setae dorsally. Costal cell with two rows of setae, ~10 dorsal setae on anterior margin apically and incomplete row of 6 ventral setae. Relative measurements:

SMV:MV:postmarginal vein:stigmal vein =26:42:22:20. Hindwing apically slightly obtuse.

**Metasoma** (Figs 1a,b,j and 2h). Petiole cylindrical, short. Gaster 1.5× as long as broad. Ovipositor sheaths with numerous setae and not protruding.

**Male**. Unknown.

**Diagnosis**. Body metallic black-green (Figs 1a,b). Scape dark. POL 2.7× as long as OOL, mouth 1.6× malar space; median carina of propodeum absent, speculum very small with sparse setations, F1 1.14× as long as F2 (Fig. 1e) and C3 with terminal spine. Forewing cubital vein not strongly curved at base, SMV tapering to apex, with 6 setae dorsally.

**Remarks**. *Diglyphus wani* sp. nov. shows some characteristics that are similar to those of *D*. *isaea*, *D*. *pulchripes*, *D*. *crassinervis*, *D*. *minoeus*, *D*. *sensilis*, and *D*.*chabrias*^14,29^. The new species is similar to *D*.*isaea*, *D*. *crassinervis*, *D*. *minoeus* and *D*. *chabrias* owing to the scape cylindrical, scape predominantly dark, femora dark brown and metallic with apical white. However, in *D*. *isaea* the speculum is absent, POL 1.4× OOL, mouth 2.0× malar space, propodeum with median carina, clava 2.0× as long as F2, and tibia metallic with base and apical 1/5–1/6 yellow, wheras in *D*. *wani* sp. nov. the speculum is very small with sparse setations, POL 2.7× OOL, mouth 1.6× malar space, propodeum without median carina, clava 2.2× as long as F2, tibia with 2/3 brown distal part. In addition, *D*. *crassinervis* and *D*. *minoeus* have the same appearance of the antennae and cubital vein (not curved at base) as *D*. *wani* sp. nov., but in *D*. *crassinervis* clava 1.1–1.2× as long as funicle, mouth almost equal to malar space, POL 1.66× OOL, speculum extending along MV, and scutellum concolorous with mesoscutum, whereas in *D*. *minoeus* mouth 1.5× or more malar space, speculum extending along parastigma, and scutellum with a purplish shine in contrast to dark metallic blue-black mesoscutum. On account of the cubital vein not curved, fore and middle tibia with basal brown markings, *D*. *wani* sp. nov. and *D*. *pulchripes* are similar. Nonetheless, in *D*. *pulchripes* the clava is as long as funicle, coxae and femora green, hind tibia brown, and base of scape white. *Diglyphus wani* sp. nov. is closed to *D*. *sensilis* owing to terminal spine of clava, absent median carina on propodeum, but differ in POL 2.25× OOL, mouth 1.33× of malar space, clava 3.8× as long as F2, and tibia with two dark bands in *D*. *sensilis*. *Diglyphus wani* sp. nov. is closed to *D*. *chabrias* with body colour, subquadrate scutellum, small speculum with sparse setations on forewing and differs in the following characters: POL 2.25× as OOL, mesoscutum 1.5× as long as scutellum, gaster 1.8× as long as broad, and fore tibia with anterior surface white and posterior surface dark and metallic or dark brown.

**Hosts and biology**. Feeding on larvae of *C*. *horticola* and *L*. *sativae*.

**Distribution**. China (Hebei, Beijing, Qinghai and Shanxi Provinces).

**Etymology**. The name of Professor Fang-Hao Wan who has made outstanding contributions to invasive biology studies in China.

#### Molecular analysis

The parasitoids only had one haplotype. The cytochrome *c* oxidase I (COI) nucleotide sequence of *D*. *wani* sp. nov. was uploaded to GenBank (accession no. MF590062). The identities of the COI sequences of *D*. *wani* sp. nov. with five similar species were 91% with *D*. *pachyneurus* and *D*. *pulchripes*, 87% with *D*. *crassinervis*, 90% with *D*. *isaea* and 85% with *D*. *minoeus*.

**Table 1:** Uncorrected *p* distance from mitochondrial COΙ partial sequences of different species between different *Diglyphus* species, distance in lower left.

Species name		Number					
		1	2	3	4	5	6
1	*Diglphus wani* sp. nov.						
2	*Diglyphus crassinervis*	0.137					
3	*Diglyphus pachyneurus*	0.093	0.137				
4	*Diglyphus pulchripes*	0.094	0.066	0.099			
5	*Diglyphus minoeus*	0.164	0.099	0.153	0.161		
6	*Diglyphus isaea*	0.101	0.136	0.098	0.102	0.113	

**Figure 3:**
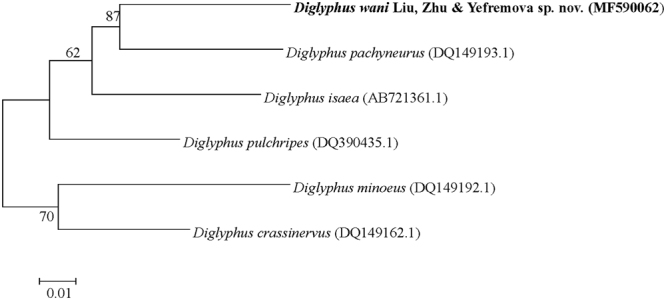
Neighbour-joining tree based on nucleotide sequence of COΙ gene of the primer COΙ SF/COΙ 2613 amplication. The access numbers which come from NCBI data base are showed in the brackets.

The percentage sequence divergence between *D*. *wani* sp. nov. and five *Diglyphus* species ranged from 9.3% with *D*. *pachyneurus* to 16.4% with *D*. *minoeus*. In addition, *D*. *sensilis* and *D*. *chabrias* were not checked with DNA sequences, because they were not found in China^14^. The uncorrected *p* distances between the different *Diglyphus* species are shown in Table 1. In the Neighbour-joining tree constructed from the COI nucleotide sequences (Fig. 3), *D*. *wani* sp. nov. clustered with *D*. *pachyneurus*.

### Life history

#### Immature development

At the beginning of the life table study, 35 eggs of *D*. *wani* sp. nov. were collected and 32 female adults emerged. The duration of the egg, larval, pre-pupal to pupal, and immature developmental periods for *D*. *wani* sp. nov. were 1.0 d, 5.0 d, 6.1 d and 12.1 d, respectively.

#### Longevity

Longevity under the host treatment was significantly longer than under the control starvation treatment (Table 2).

**Table 2:** The effects of different food treatments on life history parameters on females of *D*. *wani* sp. nov.

Treatments	Longevity (days)	No. host feeding	Parasitism	No. host stinging	Total host mortality
Host (*n* = 32)	7.7 ± 0.4a	94.4 ± 5.7	47.6 ± 2.2	3.4 ± 0.5	142.1 ± 7.0
Control (*n* = 39)	2.7 ± 0.3b	—	—	—	—
*P* value	< 0.0001	—	—	—	—

Note: Date are means ± SE. Means in columns followed by different capital letters are significantly different at *P* = 0.05. Host treatment means that parasitoid wasps are reared on late 2nd and early 3rd instar larvae of *Liriomyza sativae* (30–35 leaf^−1^) to complete three types of host-killing behaviour, whereas control treatment means that only water be provided for parasitoid wasps.

### Biocontrol potential

#### Parasitism, and host-feeding, host-stinging and host-killing events

**Figure 4:**
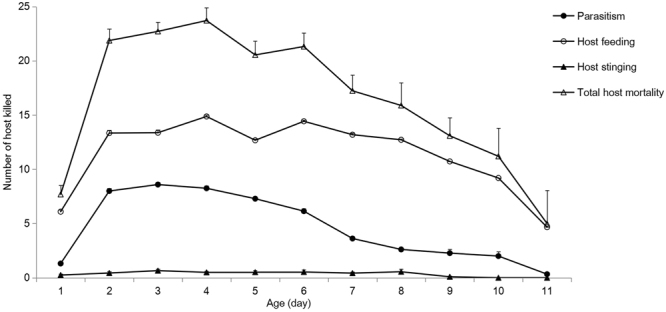
Mean (±SE) daily distribution of host mortality caused by host feeding, parasitism, host stinging of *D*. *wani* sp. nov..

**Parasitism**. In the host treatment (*n* = 32), the parasitism number was 47.6 per parasitoid wasp (Table 2) over the whole life span, whereas average daily parasitism was 6.3 hosts. The curve for the daily parasitism showed a parabolic tendency, which fluctuated slightly on the 2nd and 9th days (Fig. 4).

**Host feeding**. The parasitoids fed on 94.4 hosts during a lifetime and killed 12.2 hosts per day (Table 2). The host-feeding events accounted for 66.5% of the host-killing events. The trend in daily host-feeding events gradually increased and there was a small decrease between 4th day and 5th day. The host-feeding event was highest on the 4th day at 14.9 hosts (Fig. 4).

**Host stinging**. The number of host-stinging events was 3.4 hosts in a lifetime (Table 2), whereas the average daily host-stinging number was 0.5 hosts.

**Total host mortality**. Average of 142.1 host killings over a lifetime was observed (Table 2), with individuals killing 18.5 hosts daily. On the 1st day, the host killing was 7.7 hosts, and peaked on the 4th day, with up to 23.7 hosts were killed, and thereafter declined slowly (Fig. 4).

#### Life table

The values of *r*, *λ*, *R*_0_, and *T* of *D*. *wani* sp. nov. were 0.2373 ± 0.0046 d^−1^, 1.2678 ± 0.0061 d^−1^, 43.2 ± 3.1 offspring, and 15.0 ± 0.2 d, respectively.

#### Consumption rate of three host-killing behaviour

**Figure 5:**
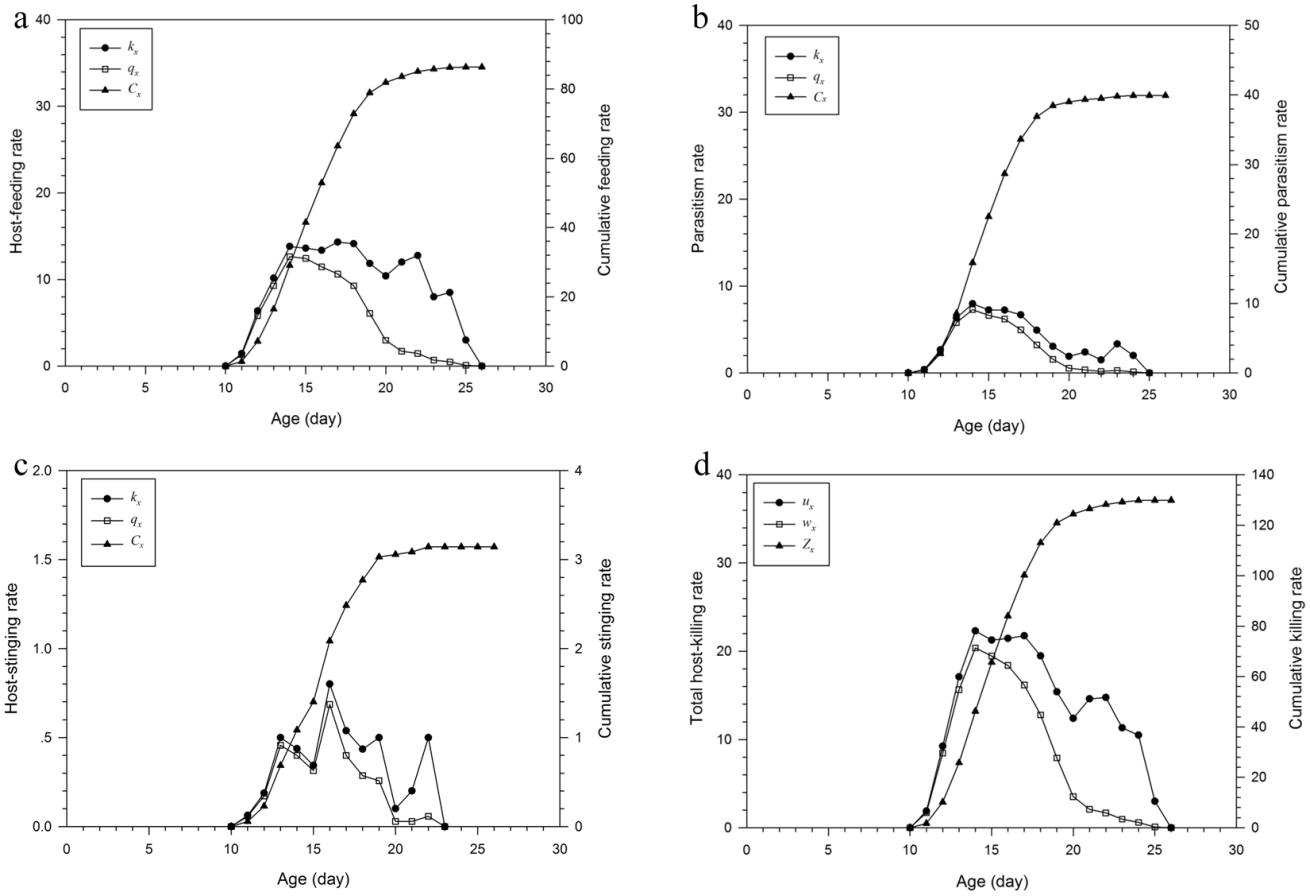
(**a**) Age-specific host-feeding rate (*k*_*x*_), net age-specific host-feeding rate (*q*_*x*_), and cumulative host-feeding rate (*C*_*x*_) of *D*. *wani* sp. nov. (**b**) Age-specific parasitism rate (*k*_*x*_), net age-specific parasitism rate (*q*_*x*_), and cumulative parasitism rate (*C*_*x*_) of *D*. *wani* sp. nov. (**c**) Age-specific host-stinging rate (*k*_*x*_), net age-specific host-stinging rate (*q*_*x*_), and cumulative host -stinging rate (*C*_*x*_) of *D*. *wani* sp. nov. (**d**) Age-specific total host-killing rate (*u*_*x*_), net age-specific total host-killing rate (*w*_*x*_), and cumulative total host-killing rate (*Z*_*x*_) of *D*. *wani* sp. nov.

**Host-feeding rate**. There was no consumption during the egg, larval, and pre-pupal to pupal stages, hence we calculated the host-feeding rate using the host feeding of adults. Thus, there was a blank stage before the females emerged. The maximum daily host-feeding rate of *D*. *wani* sp. nov. was 14.3 hosts on the 17th day (Fig. 5a). The net daily host feeding was 12.6 hosts on the 14th day (Fig. 5a). The *C*_0_, *ψ*, *ω* and *Q*_q_ values were 86.3429 ± 6.8367, 0.5143 ± 0.033, 0.6520 ± 0.0442, and 1.9974 ± 3.0821, respectively.

**Parasitism rate**. The age-specific and net age-specific parasitism rate curves of *D*. *wani* sp. nov. initially increased gradually. After the 14th day, the curves decreased with increasing age (Fig. 5b). The net age-specific parasitism rate peaked at 8 hosts on the 14th day (Fig. 5b). The *C*_0_, *ψ*, *ω*, and *Q*_q_ values were 39.9143 ± 2.8275, 0.2575 ± 0.0172, 0.3264 ± 0.0233 and 0.9233 ± 0.0116, respectively.

**Host-stinging rate**. The maximum daily age-specific host-stinging rate was 0.8 hosts at 17th day of age (Fig. 5c). Both the daily age-specific and net age-specific host-stinging rate curves gradually increased with fluctuations prior to 17th day and then declined dramatically (Fig. 5c). The *C*_0_, *ψ*, *ω* and *Q*_q_ values were 3.1429 ± 0.5154, 0.0193 ± 0.0029, 0.0244 ± 0.0037 and 0.0727 ± 0.0109, respectively.

**Total host-killing rate**. The total host-killing rate was the sum of the parasitism, host-feeding and host-stinging rates. The value of *Z*_*x*_ showed an upward trend. The *p*_*x*_ and *u*_*x*_ values peaked at 20.4 and 22.3 hosts, respectively, at 14th day of age (Fig. 5d). Thereafter, *p*_*x*_ and *u*_*x*_ declined with increasing age. The *C*_0_ value of the wasp on *L*. *sativae* was 129.9100 ± 9.2230 leafminers per individual. The *ψ* and *ω* values were 0.7944 ± 0.0472 and 1.0071 ± 0.0642, respectively. The *Q*_q_ value of the wasp was 3.0053 ± 0.1112 leafminers for the production of each egg.

### Ovigeny index

The number of mature eggs in newly emerged females was 0.72 ± 0.25 (*n* = 32). The parasitoids produced 47.6 eggs during the whole life span. Thus, the ovigeny index was 0.015.

### The gains in longevity and fecundity per host-feeding event

The starved parasitoids had the greatest egg load (2.7 eggs, *n* = 24) at 12 h. The parasitoid wasps fed one larva produced 0.5 eggs and had an increased longevity of 0.1 d.

### Relationships between host feeding and longevity, parasitism, host stinging, and total host mortality

**Figure 6:**
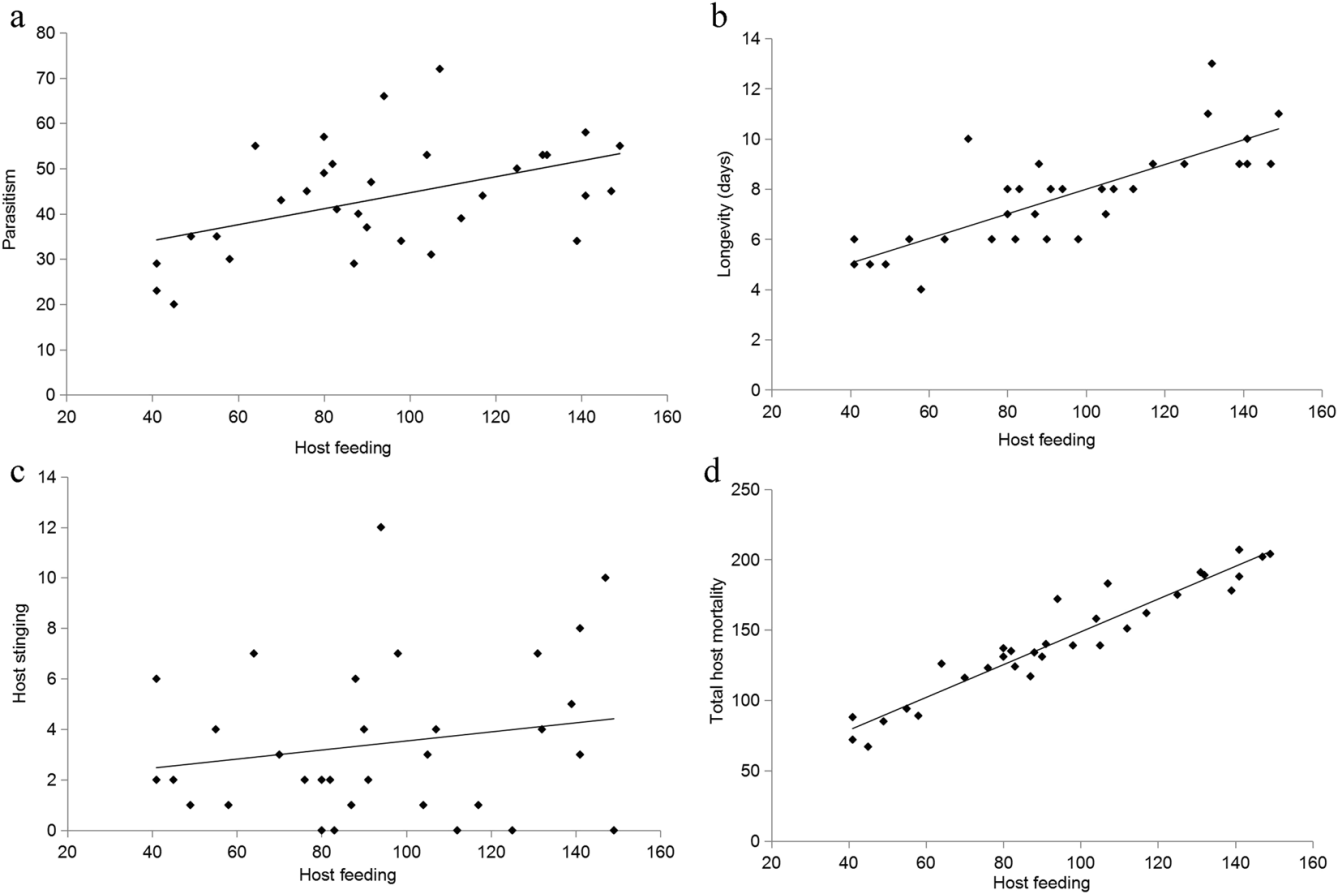
Relationships between the number of host-feeding events by *D*. *wani* sp. nov. and parasitism (**a**), longevity (**b**), host-stinging events (**c**) and total host mortality (**d**). Lines represent linear regressions.

Host feeding was strongly correlated with longevity (*y* = 0.0491*x* + 3.0796, r^2^ = 0.6149, *F*_*1–32*_ = 47.89, *P* < 0.0001, Fig. 6a), parasitism (*y* = 0.1765*x* + 26.986, r^2^ = 0.2252, *F*_*1–32*_ = 8.72, *P* = 0.0061, Fig. 6b), and total host mortality (*y* = 1.1651*x* + 32.0680, r^2^ = 0.9152, *F*_*1–32*_ = 323.87, *P* < 0.0001, Fig. 6d). However, host feeding was not correlated with host stinging (*y* = 0.0179*x* + 1.7443, r^2^ = 0.0358, *F*_*1–32*_ = 1.11, *P* = 0.2996, Fig. 6c).

## Discussion

Based on distinct morphological characteristics and mitochondrial COI sequence analyses, the species was named *Diglyphus wani* Liu, Zhu & Yefremova sp. nov. To our knowledge, this is the first thelytokous species described in the genus *Diglyphus*. This wasp is a numerically dominant member of the parasitoid assembly of agromyzid leafminers in fields in Qinghai, Hebei, Beijing and Shanxi Provinces, China (unpublished data).

The thelytokous parthenogenesis of this wasp, in which females produce daughters without mating, was observed. So far, the males of *D*. *wani* sp. nov. are absent in the field or in the laboratory colonies. According to the review by Ma and Schwander^26^, female-producing parthenogenesis arises by three main evolutionary mechanisms. First, thelytokous parthenogenesis could be induced by endosymbionts that increased their transmission by manipulating host reproduction, which was considerated to be a main route. *Wolbachia*, *Cardinium* and *Rickettsia* genera of parthenogenesis-inducing bacteria occured in Hymenoptrea. Endosymbiont-induced parthenogenesis that was known or suspected in 124 host species from seven arthropod taxa, of which *Wolbachia* was the most frequent endosymbiont (in 56–75% of host species)^26^. Stouthamer^30^ indicated that at least 270 hymenopteran species showed female-producing parthenogenesis. Among Hymenoptera, *Cardinium* bacteria were known to occur only in chalcidoid wasps. At present, thelytokous reproduction caused by a parthenogenesis-inducing bacterium *Rickettsia* is known only in two hymenopteran parasitoids, *Neochrysocharis formosa* and *Pnigalio soemius*, and one hymenopteran pest, *Leptocybe invasa*^31–33^. Endosymbiont-induced parthenogenesis appears to facilitate the maintenance of reproductive polymorphism, with at least 50% of species consisting of both thelytokous (infected) and arrhenotokous (uninfected) strains^26^. Second, thelytokous colony can arise as spontaneous mutations, as has occurred in, *Daphnia* water fleas^34^ and monogonont rotifers^35^. However, it is rare in the Hymenoptera. Finally, hybridization between two sexually reproducing species can induce transformation of the sexual reproduction into thelytokous parthenogenesis, however, this phenomenon appears to happen frequently among vertebrates. In addition, the mechanisms of thelytokous parthenogenesis in some species are not clear (like thelytokous strain of *Venturia canescens*^36^ and *Aptinothrips rufu*^26^). Given that the laboratory-reared colony of *D*. *wani* sp. nov. about 55 generations did not produce males under laboratory conditions and no arrhenotokous strain of *D*. *wani* sp. nov. was found in the field. These results showed that *D*. *wani* sp. nov. was a thelytokous or female-producing parthenogenetic species rather than a cyclical parthenogenetic organism. Although *Rickettsia* was detected in four individuals among ten individuals selected from the original colony (210 individuals), females only produced daughters from F1 to F5 generations under antibiotic treatment (20 mg/ml tetracycline) (unpublished data). The cause of female-producing parthenogenesis of *D*.*wani* sp.nov. need to study further in the future.

*Diglyphus wani* sp. nov. is indicated to be a parasitoid with three types of host-killing behaviour. All host-killing parasitoids show host-feeding behaviour. More than 140 species from 17 families of Hymenoptera feed on their hosts after adult eclosion^37^. The total number of host-feeding parasitoid species has been estimated at almost 100,000, or about one-third of all parasitoid species^38^. Recently, additional host-feeding parasitoid species, especially eulophids, that attack leafminers have observed to exhibit three types of host-killing behaviour^13,19–24^. However, the host-killing rates vary in different parasitoids, such as *D*. *isaea*, *N*. *formosa*, *Hemiptarsenus varicornis*, and *P*. *soemius*^13,18,23,24^. These species have been widely studied and all are common parasitoid wasps against leafminers. The proportions of parasitism, host feeding and host stinging among the host-killing events of *D*. *wani* sp. nov. were 30.7%, 66.5% and 2.8%, respectively. For the ectoparasitoid *H*. *varicornis*, the proportions of the three behaviour were 25.7%, 58.2% and 16.1%, respectively, whereas for the endoparasitoid *N*. *formosa* the proportions were 44.4%, 31.1% and 24.6%, respectively^13,18,23,24^. These results may reflect a trade-off strategy, which is determined by the decision whether to invest in current or future reproduction of insect parasitoids^39^.

In addition, all host-feeding parasitoids studied to date are synovigenic^18,19,21,24,40^. The female wasps must feed on host tissues and host haemolymph to promote continuous oogenesis and egg maturation because most adult parasitoids cannot generate lipids *de novo*^41,42^. As a host feeder, *D*. *wani* sp. nov. is also strongly synovigenic. Furthermore, such parasitoids feed on hosts to increase their longevity^13,19,24,43^, as does *D*. *wani* sp. nov. The present results and previous results show that the host-feeding behaviour of these parasitoids have significant strongly positive linear relationships with longevity and fecundity^13,18,19,24^. However, the values of single host-feeding behaviour in different parasitoids were diverse. For example, when a female *D*. *wani* sp. nov. generated a host-feeding behaviour, the fecundity and longevity increased by 0.5 eggs and 0.1 d, respectively. However, Cheng *et al*.^13^ found that when a female *H*. *varicornis* fed on a host *L*. *trifolii*, it increased the fecundity by 0.2 eggs and the longevity by 0.1 d. Both *D*. *isaea* and *N*. *formosa* that fed on a host *L*. *sativae* increased their fecundity levels by 2.6 and 1.4 eggs, respectively, and their longevities by 0.4 d and 0.2 d, respectively^18^. All of these species are widely used to control leafminers. Variation in the contributions of host-feeding behaviour among these species may be dependent on differences in the nutrient accumulation and utilisation capacities of diverse parasitic wasps^19,44,45^.

Compared with the results of Zhang *et al*.^19^, in which *D*. *isaea* reared on *L*. *sativae* at 25 °C, the values for parasitism, host stinging, host mortality, and longevity of over the whole lifespan were lower. However, the daily total host mortality (18.5 hosts d^−1^) and the average daily fecundity (6.3 eggs d^−1^) of *D*. *wani* sp. nov. were greater than those of *D*. *isaea* (12.6 hosts d^−1^ and 2.6 eggs d^−1^, respectively)^18^. In addition, *D*. *wani* sp. nov. showed a higher daily reproductive ability than *D*. *begini* or *D*. *isaea*, whereas the number of hosts parasitised per day for *D*. *wani* sp. nov. was lower than that of *D*. *intermedius*^18,46,47^. When the overall number of host-feeding events of *D*. *wani* sp. nov. (94.4 hosts female^−1^) was higher compared with those of other parasitoids that attack leafminers, such as *D*. *isaea* and *N*. *formosa* cultured from *L*. *sativae* with means of 56.6 and 43.7 hosts female^−1^, respectively^18^. In contrast, the host-stinging to total mortality ratio of *D*. *wani* sp. nov. was less than those of other parasitoids. For instance, the host stinging-to-total mortality of *D*. *isaea* and *P*. *soemius* attained 39.9% and 52.0%, respectively^19,23^. Patel *et al*.^47^ suggested that the proportions of different host-killing events depended on the density of leafminer larvae on individual leaflets.

Host-killing parasitoids may show greater biocontrol potential than parasitoids capable of only reproductive parasitisation owing to their strong destructive non-reproductive host-killing capacities^13,18,20,24,37,48^. For instance, in the present study, the non-reproductive rate of *D*. *wani* sp. nov. was 69.3%. Given the non-reproductive behaviour of host-killing parasitoids, the assessments of their biocontrol abilities differ from other parasitoid wasps that only kill hosts by parasitism. Thus, numerous attempts have been made to determine the biocontrol potential of host-feeding parasitoids that attacked the same hosts. In addition, to assess the population growth potential and the efficacies of the biocontrol potential, we determined an age-specific survival rate and finite host-killing rate. For the parasitoid wasp *D*. *wani* sp. nov., the intrinsic rate of increase, net reproductive rate, and finite host-killing rate were high, whereas the mean generation time was short. The present study illustrated that the short developmental duration, thelytokous reproduction and three host-killing behaviour render *D*. *wani* sp. nov. to a good natural enemy for the biological control of leafminers.

Although *D*. *isaea* is considered to show good biocontrol potential and broad application prospects, the species is easily prone to sex ratio imbalances in artificially generated rearing processes that affect its application^7^. Thelytokous wasps show a higher rate of population increase because individuals do not mate and waste eggs^26,27^. Therefore, *D*. *wani* sp. nov. might be a favourable biocontrol agent against agromyzid leafminers.

Temperature is a factor that commonly influences parasitoid life-history traits and biocontrol potential. Despite the distribution of *Diglyphus* in the Holarctic region and its adaptation to a cool climate, some parasitoid species in the genus may be used in warm environments^7^. In the field, parasitoids can simultaneously rely on hosts and non-host nutrient sources, such as floral tissues, hemipteran honeydew and pollen, and various artificial diets have been used in mass rearing^43^. To provide insights into the development of integrated control strategies for leafminer pests in field- and greenhouse-grown crops, the temperature-related adaptability of thelytokous parasitoids and the impact of adult diet on parasitoid reproduction require further study.

## Methods

### Insect cultures

The parasitoids (210 individuals) used in this study were originally collected from leafminer *C*. *horticola* infesting leaves of *P*. *sativum* in Xining, Qinghai, China (36°43′23.52″N, 101°45′1.51″E) in July 2015. This colony from one line was reared on *Phaseolus vulgaris* leaves infested with *L*. *sativae* larvae and supplemented with 20% honey water in gauze cages over 15 generations. The colony of *L*. *sativae* was obtained from *P*. *vulgaris* in Langfang, Hebei, China (39°35′14.77″N, 116°47′37.27″E) and cultured for 20 generations on *P*. *vulgaris* supplemented with 20% honey water. The leafminers and parasitoid wasps were maintained in climate chambers set to 25 ± 1 °C, relative humidity of 30 ± 5%, and a photoperiod of 14:10 h (light:dark). All tests were performed in climate chambers at the State Key Laboratory for Biology of Plant Diseases and Insect Pests, Institute of Plant Protection, Chinese Academy of Agricultural Sciences (Beijing, China).

### Species identification

#### Morphology diagnosis

The parasitoids used for morphological identification were collected from field populations (Qinghai: 5♀; Shanxi: 2♀; Hebei: 4♀; Beijing: 1♀) and the laboratory colony (5♀). The parasitoid specimens were killed in 99.7% EtOH and preserved at −80 °C for the taxonomic study. Specimens were examined with a stereomicroscope (Olympus Corporation, SZX-16, Tokyo, Japan).

The morphological terminology followed that used by Gibson^49^. Abbreviations used are: F1–F2, first through second flagellomeres; SMV and MV, submarginal and marginal, veins, respectively; OOL, minimum distance between an eye margin and the adjacent posterior ocellus; and POL, minimum distance between the posterior ocelli.

Measurements in millimetres (mm) of body and forewing lengths were recorded using a stereomicroscope (Olympus Corporation, VHX-2000, Tokyo, Japan). For all other dimensions, relative measurements were used. Photographs of the species were taken using an Olympus CX31 microscope with a Helicon Focus system. Pictures were taken of critical-point-dried specimens (30 min after emergence) using a scanning electron microscope (Hitachi Corporation, SU8010, Tokyo, Japan) at an acceleration voltage of 10.0 kV.

#### Molecular diagnosis

Individual wasps from the laboratory colony were randomly chosen and killed by freezing at −80 °C for 10 min. The each body of an individual was used for DNA extraction following the method described by De Barro and Driver^50^.

The COI gene (744 bp) was amplified using the primers COI SF^51^ and COΙ 2613^52^. All PCR reactions were carried out in a standard 25 μl volume, using 0.25 μl Taq DNA polymerase (2.5 U μl^−1^), 0.5 μl dNTPs (2.5 mM each), 2.5 μl 10× buffer (+Mg^2+^), 0.5 μl forward primer, 0.5 μl reverse primer, 2 μl template DNA and 18.75 μl dd H_2_O. The PCR protocols for COI amplification followed those reported by Sha *et al*.^51^. Amplification was performed using an ABI thermal cycler (Veriti™ Applied Biosystems 9902, Singapore).

After a positive PCR amplification, PCR products were purified and sequenced by Sangon, Shanghai, China.

Forward and reverse strands were first aligned using MEGA 7.0^53^ with default parameters. The sequences were aligned in the NCBI BLAST database. The other sequences for other *Diglyphus* species (such as *D*. *isaea*, *D*. *pachyneurus*, *D*. *pulchripes*, *D*. *minoeus* and *D*. *crassinervus*), which show similar morphological features, were downloaded from NCBI for phylogenetic, sequence divergence and haplotype analyses using MEGA 7.0 and DNA sp 5.10.01^54^. Phylogenetic tree reconstruction was implemented using the neighbour-joining approach based on the uncorrected “*p*” distances model in MEGA 7.0. A bootstrap analysis with 1,000 replicates was performed to assess branch support.

### Life history and biocontrol potential

#### Immature development

To obtain data on immature development, kidney bean leaves infested with late 2nd and early 3rd instar *L*. *sativae* larvae (30–35 leaf^−1^) were cut from the plants. To maintain the leaves fresh, 10 ml water–agar (1%) was trickled into a petri dish. After refrigeration in the lab, the leaves were individually and immediately placed on the agar gel surfaces in the petri dishes and covered with cling film pricked with a dissecting needle to allow air circulation. Then, 5–10 parasitoid wasps (3 d after emergence) obtained from the laboratory colony were placed in each petri dish with 20% honey water to collect parasitoid eggs. After 3 h, the parasitoid wasps were removed and petri dishes with both parasitised and unparasitised leafminers were maintained in a climate chamber set at 25 ± 1 °C, relative humidity of 50 ± 5%, and a photoperiod of 14:10 h (light:dark). In total, 35 eggs were used at the beginning of the immature development study. Eggs were individually recorded and checked at different stages using a stereomicroscope every 12 h until adult emergence. The prepupae were placed individually in vials, which were tightly sealed with cotton to maintain moisture, for rearing in the laboratory and the immature development of *D*. *wani* sp. nov. was recorded every 6 h.

#### Adults life history: longevity and three host-killing behaviour

Newly emerged parasitoid wasps (08:00–10:00) of uniform body length were randomly assigned to the control (starvation treatment, *n* = 30) and host (*n* = 32) treatments under the above mentioned conditions in a climate chamber. One adult was added to each petri dish. In the control, the wasps were only allowed to feed on water, and the longevity was recorded and checked every 12 h (08:00 and 20:00). In the host treatment, one female was introduced to a petri dish containing one leaf infested with 30–35 late 2nd and early 3rd instar larvae of *L*. *sativae*, and allowed to implement host-killing behaviour for 24 h. Each petri dish was renewed daily until the wasp died. Meanwhile, the treated petri dish was placed in the same climate chamber as mentioned above. After 48 h, eggs or larvae of parasitoid wasps were observed under a stereomicroscope, and the three types of host-killing behaviour (host feeding, parasitism, and host stinging) were identified using the method of Cheng *et al*.^13^. The longevity, host feeding, parasitism, host stinging, and host mortality were recorded every 24 h.

#### Ovigeny index

To describe the relationship between ovigeny and life history, Jervis *et al*.^22^ devised a quantitative “Ovigeny index” that was calculated as the proportion of the maximum potential lifetime complement that is mature upon female emergence. In synovigenic species the index value is less than 1, whereas in pro-ovigenic species the value is 1^22^.

From among the newly emerged parasitoid wasps (08:00–8:30) that were reared on late 2nd and early 3rd instar larvae of *L*. *sativae* in an artificial climate chamber, 32 individuals were randomly selected and frozen for 2 min at −80 °C. Subsequently, the wasps were dissected on a microscope slide with PBS buffer (pH = 7.2), and the initial eggs were counted under a stereomicroscope. The real lifetime fecundity data were obtained from the above section, and the initial egg loads were used to calculate the ovigeny index value.

#### Gains in longevity and fecundity per host-feeding event

Based on previous methods, we estimated the number of mature eggs and the number of days added to the lifespan per host-feeding event for a given female^13,18,19,37^. To obtain the greatest number of mature eggs, we compared different starvation treatments (6, 12, 24, and 48 h; *n* = 5 for each) for females only allowed distilled water. After the 12 h starvation treatment, 32 females were dissected and the number of mature eggs recorded because this group had the greatest number of mature eggs in a preliminary experiment. The longevity of females in the control was determined as described in the preceding section.

#### Life table

According to methods of the age-stage, two-sex life table^54–56^, the life history raw data were analysed using the TWOSEX-MSChart software^57^. Life table parameters, such as intrinsic rate of increase (*r*), finite rate of increase (*λ*), net reproductive rate (*R*_0_) and mean generation time (*T*), were calculated in accordance with the previous studies.

#### Finite host-killing rate

To analyse the host-feeding, parasitism, host-stinging, and total host-killing rates, we followed the methods of Wang^58^ using the CONSUME-MSChart software^59^ with slight modifications.

The age-specific consumption (host feeding/parasitism/host stinging) rate (*k*_*x*_) represents the number of hosts killed by the parasitoids of individuals at age *x* and was calculated according to the following equation:

1 $${k}_{x}=\frac{{\sum }_{j=1}^{\beta }{s}_{xj}{c}_{xj}}{{\sum }_{j=1}^{\beta }{s}_{xj}}$$

where *c*_*xj*_ is the age-stage-specific host-killing (host feeding/parasitism/host stinging) rate of individuals at age *x* and life stage *j*, whereas *s*_*xj*_ is the age-stage-specific survival rate of individuals at age *x* and stage *j*. When the age-specific survival rate (*l*_*x*_), which is the number of individuals that survive to age *x*, was taken into consideration, the net age-specific host-killing (host feeding/parasitism/host stinging) rate (*q*_*x*_) was calculated as follows:

2 $${q}_{x}={l}_{x}{k}_{x}$$

The cumulative host-killing (host feeding/parasitism/host stinging) rate (*C*_*x*_) is defined as the number of hosts killed by a parasitoid from birth to age *x* and it was calculated as follows:

3 $${C}_{x}=\sum _{i=0}^{x}{l}_{i}{k}_{i},{C}_{0}=\sum _{x=0}^{{\rm{\infty }}}{l}_{x}{k}_{x}=\sum _{x=0}^{{\rm{\infty }}}\sum _{j=1}^{{\rm{\infty }}}{s}_{xj}{c}_{xj}$$

The stable host-killing (host feeding/parasitism/host stinging) rate (*ψ*) represents the proportion of individuals belonging to age *x* and stage *j* in a stable age-stage distribution (*ɑ*_*xj*_):

4 $$\psi =\sum _{x=0}^{\infty }\sum _{j=1}^{m}{a}_{xj}{c}_{xj}$$

To assess the host-killing (host feeding/parasitism/host stinging) potential, the finite host-killing (host feeding/parasitism/host stinging) rate (*ω*) was calculated as follows:

5 $$\omega =\lambda \psi $$

The total host-killing rate (*u*_*x*_) represents the total number of host feeding, parasitism and host stinging events, as follows:

6 $${u}_{x}=\frac{{\sum }_{j=1}^{\beta }{s}_{xj}{p}_{xj}}{{\sum }_{j=1}^{\beta }{s}_{xj}}$$

The age-stage-specific total host-killing rate of individuals at age *x* and stage *j* (*p*_*xj*_) was the summation of *c*_*xj*_ of host feeding, *c*_*xj*_ of parasitism and *c*_*xj*_ of host stinging, respectively:

7 $${p}_{xj}={c}_{xj(hostfeeding)}+{c}_{xj(parasitism)}+{c}_{xj(hoststinging)}$$

The net age-stage-specific total host-killing rate (*w*_*x*_) was calculated as follows:

8 $${w}_{x}={l}_{x}{u}_{x}$$

The net total host-killing rate (*Z*_0_) represents the total number of three host-killing events during one generation of the wasps. It was calculated as:

9 $$\,{Z}_{0}=\sum _{x=0}^{\infty }\sum _{j=1}^{\beta }{s}_{xj}{p}_{xj}$$

The cumulative total host-killing rate (*Z*_*x*_) represents the total number of hosts killed by a parasitoid wasp from birth to age *x*. It was calculated as:

10 $$\,{Z}_{x}=\sum _{i=0}^{x}{l}_{i}{u}_{i}$$

To compare the overall host-killing potential, the finite total host-killing rate (*θ*) and the stable total host-killing rate (*ϑ*) were as calculated as follows:

11 $$\,\theta =\lambda \vartheta $$

12 $$\,\vartheta =\sum _{x=0}^{\infty }\sum _{j=1}^{m}{a}_{xj}{p}_{xj}$$

The transformation rate from host population to parasitoid offspring (*Q*_P_) is the mean number of hosts a parasitoid needs to kill to produce one offspring. It is calculated as:

13 $${Q}_{{\rm{p}}}=\frac{{Z}_{0}}{{R}_{0}}$$

### Data analyses

Data on the three types of host-killing behaviour were analysed with a one-way ANOVA using Fisher’s model under the 0.05 level of significance. Linear regression was used to assess the relationships between host feeding and longevity, parasitism, host stinging and host mortality.
